# Endophyte *Chaetomium globosum* D38 Promotes Bioactive Constituents Accumulation and Root Production in *Salvia miltiorrhiza*

**DOI:** 10.3389/fmicb.2017.02694

**Published:** 2018-01-22

**Authors:** Xin Zhai, Dong Luo, Xiuqing Li, Ting Han, Min Jia, Zhouyang Kong, Jiachen Ji, Khalid Rahman, Luping Qin, Chengjian Zheng

**Affiliations:** ^1^Department of Pharmacognosy, School of Pharmacy, Second Military Medical University, Shanghai, China; ^2^Faculty of Science, School of Biomolecular Sciences, Liverpool John Moores University, Liverpool, United Kingdom

**Keywords:** *Chaetomium globosum*, endophytic fungus, *Salvia miltiorrhiza*, secondary metabolism, plant growth

## Abstract

*Salvia miltiorrhiza* is known for tanshinones and salvianolic acids, which have been shown to have a protective effect against ROS, especially for cardiovascular diseases and other various ailments of human organs. Due to the low yield of tanshinones and their analogs in *S. miltiorrhiza*, multiple stimulation strategies have been developed to improve tanshinones production in plant tissue cultures. Endophytic fungi have been reported to form different relationships with their host plants, including symbiotic, mutualistic, commensalistic, and parasitic interactions. Thus we take the assumption that endophytic fungi may be a potential microbial tool for secondary metabolism promotion in medicinal plants. We recently isolated *Chaetomium globosum* D38 from the roots of *S. miltiorrhiza* and our study aimed to examine the effects of this live endophytic fungus D38 and its elicitor on the accumulation of tanshinones in the hairy root cultures of *S. miltiorrhiza*. Our results revealed that *C. globosum* D38 mainly colonized in the intercellular gap of xylem parenchyma cells of *S. miltiorrhiza* hairy roots during the long term co-existence without any toxicity. Moreover, both of the live fungus and its mycelia extract could increase the production of tanshinones, especially for dihydrotanshinone I and cryptotanshinone. The effect of the mycelia extract was much stronger than that of the live fungus on tanshinones synthesis, which significantly increased the transcriptional activity of those key genes in tanshinone biosynthetic pathway. Furthermore, the live *C. globosum* D38 could also be made into biotic fertilizer used for *S. miltiorrhiza* seedlings culture, which not only significantly promoted the growth of the host plant, but also notably enhanced the accumulation of tanshinones and salvianolic acids. We thus speculated that, in the soil environment D38 could form bitrophic and mutual beneficial interactions with the host and enhance the plant growth and its secondary metabolism on the whole so as to have facilitative effects on both tanshinones and salvianolic acids accumulation. In conclusion, *Chaetomium globosum* D38 was a highly beneficial endophytic fungus for the growth and metabolism of *S. miltiorrhiza*.

## Introduction

Herbal medicines consumption has been increasing steadily and plant-derived secondary metabolites have become an important part for human health and nutrition. However, the yield of secondary metabolites in plants is limited and depends greatly on physiological stages of the plants (Chandra and Chandra, 2011). In recent years, plant tissue cultures have become a promising alternative source for bioactive secondary metabolites increasingly desired in medical and pharmaceutical field (Ahlawat et al., 2014). As the most convenient and useful experimental material, plant tissue cultures are also usually used to explore and investigate the effects of various biological measures on desired products biosynthesis and production (Jian et al., 2005; Kai et al., 2011).

It is known that elicitation can enhance the biosynthesis of secondary metabolites and biotic elicitors (Thwe et al., 2016) have particularly been used to induce the secondary metabolites accumulation in medicinal plants (Bahabadi et al., 2014). Therefore, it is one of the most effective means to stimulate secondary metabolites biosynthesis in plant tissue cultures, such as hairy roots and plant cells, using pathogenic and nonpathogenic fungi as biotic elicitors (Huang et al., 2016). Generally, fungi can easily switch from endophytic to necrotrophic lifestyle at the evolutionary and even ecological timescale. Different from necrotrophic fungi, the endophytes could infect host tissues without any concurrent symptoms of disease (Krings et al., 2007). In many cases, endophytes form mutualistic interactions and beneficial relationship with their host plants (Hyde et al., 2008; Rodriguez et al., 2009), which can not only stimulate plant growth, but also promote secondary metabolites accumulation in the plant (Murthy et al., 2014). However, not all the endophytes have the ability to co-culture with their host plant tissues for a long time, which depends on the toxicity of the fungal isolate. In most cases, those fungi were fabricated into elicitors with their toxicity removed, which can also stimulate the secondary metabolism of the host plants (Giauque and Hawkes, 2013). So fully elucidation of above-mentioned host–microbe interactions, may provide solid evidence for the favorable effects of long-term microbial beneficially colonization on herbal plants (Guo et al., 2015).

*Salvia miltiorrhiza* Bunge (Lamiaceae) is a very famous and important medicinal plant in China and its roots have been traditionally utilized to treat cardiovascular, menstrual and various inflammation-related diseases (Han et al., 2008; Wu and Yeung, 2010). Tanshinones are a series of bioactive phenanthrenequinones in *S. miltiorrhiza* roots, which demonstrates versatile pharmacological activities (Yoon et al., 1999; Yin et al., 2008; Lee et al., 2010), including antioxidant, cardiovascular protective, antibacterial, anti-inflammatory and antineoplastic activities (Tu et al., 2012; Koji et al., 2015). However, the low yield of tanshinones usually requests the use of large amount of plant material and thus appears as a major obstacle for *S. miltiorrhiza* exploit (Kai et al., 2012).

Although treatment with elicitor is one of the most effective means for stimulating secondary metabolism in medicinal plants (Huang et al., 2016), there have been few studies documented on the effects of elicitors, such as yeast extract (YE), salicylic acid (SA), and methyl jasmonate (MJ), on tanshinones metabolism in beneficial *S. miltiorrhiza* hairy root (Hui et al., 2001; Ge and Wu, 2005). Only our group has previously reported an elicitor from endophyte *Trichoderma atroviride* D16, which could significantly promote the biosynthesis of tanshinone constituents (Ming et al., 2013). However, the hairy roots of *S. miltiorrhiza* can not be long-term co-cultured with *T. atroviride* D16. So our group continued to search for other favorable endophytes, which could enhance tanshinones production without any toxicity against *S. miltiorrhiza* hairy root during long-term co-culture. The present study is therefore the first report on the effects of a live endophytic fungus and its elicitor on the tanshinones production in *S. miltiorrhiza* hairy root. In this study, the tanshinones-promoting endophyte was identified as *Chaetomium globosum* D38. *C. globosum* has been recognized as an effective biocontrol fungus especially in agriculture (Reissinger et al., 2003), which could enhance seedlings tolerance to stress (Cullen et al., 1984; Abou Alhamed and Shebany, 2012). The effects of *C. globosum* D38 and its extract of mycelium (EM) on the tanshinones accumulation in *S. miltiorrhiza* hairy root cultures were further studied and the possible mechanism was also investigated for better understanding the role of *C. globosum* D38 in host-microbe interactions. Additionally, *Chaetomium globosum* D38, processed as a biotic fertilizer, was also administrated to *S. miltiorrhiza* seedlings to test whether this live fungus could exert favorable effects on the whole plant so as to lay the foundation for further practical applications.

## Materials and Methods

### Isolation and Identification of Endophytic Fungus D38

First of all, in order to remove soil and dirt, the roots of *S. miltiorrhiza* (collected in Shanluo, Shanxi, China; voucher specimen: #20140504) were washed by running water, followed by deionized water. According to the literature (Huang et al., 2009; Tan et al., 2012), the root was cut into 0.5 cm section and sterilized successively by 75% ethanol for 30 s, 2.5% sodium hypochlorite for 60 s, and 75% ethanol for 30 s. Then, they were rinsed by sterile water for four times and desiccated by sterile filter paper. Finally, the tissues were placed and cultured on PDA medium contained 100 mg L^-1^ penicillin at 28°C. The new hyphal was transferred separately on new medium and incubated subsequently at 28°C for 14 days. This process was repeated until we obtained a pure strain.

The purified endophytic fungus was cultured on PDA medium at 28°C for 7 days and was characterized by morphological features. The mycelium was scraped from the surface of the PDA medium and its genomic DNA was extracted by CTAB method (Cota-Sánchez et al., 2006). The ITS regions and 5.8 S gene were amplified by using the universal primers ITS5 (5′-GGAAGTAAAAGTCGTAACAAGG-3′) and ITS4 (5′-TCCTCCGCTTATTGATATGC-3′) (Min, 2013), which were finally compared by BLAST search at the GenBank and aligned with CLUSTAL X software by using 1000 bootstrap replicates (Larkin et al., 2007). The phylogenetic tree was performed using the neighbor-joining method with MEGA7 software based on Kimura 2-parameter model and identification at species taxonomic levels was based on ≥ 99% ITS similarity (Achille et al., 2006). The GenBank accession number of the nucleotide sequence was MF461354 and the endophytic fungus D38 has been kept in China General Microbiological Culture Collection Center in Beijing, China (collection number CGMCC 12622).

### Hairy Root Culture

*Salvia miltiorrhiza* hairy roots used in our work were induced and obtained by infecting the plantlets with a Ri T-DNA-bearing *Agrobacterium rhizogenes* bacterium (C58C1) (Ming et al., 2013). The hairy root was inoculated to 250 mL conical flasks containing 100 mL 1/2 B5 medium, which were then cultured at 25°C, 135 rpm in darkness for 3 weeks. The medium was changed once every week and hairy roots were randomly divided into control group and experimental groups. The plaque of 5 mm diameter was inoculated with an inoculation loop to conical flask as experimental groups, while the same size of medium patch was inoculated into the control group. Endophytic fungi D38 was cultured with hairy roots for 18 days, which were sampled on 0, 6, 12, and 18 days respectively. Each treatment has three repeats at each time for every group and nutrient solution was changed every 6 days.

### Preparation of D38 Elicitor from the Extract of Mycelium

The plaque of D38 in PDA medium (5 mm in diameter) was inoculated to 250 mL conical flask. The conical flasks contained about 100 mL 1/2 B5 medium, and were cultured at 25°C, 135 rpm in the table concentrator. After 7 days, D38 culture broth was filtrated, with D38 mycelium retained. After washed five times by distilled water, D38 mycelium were suspended in distilled water, homogenated for 5 min and ultrasonically extracted for 60 min. Then, D38 mycelium solution was put at 121°C for 30 min in a high pressure steam sterilization pot to remove free protein in this polysaccharide solution, which was subsequently filtered through three pieces of filter paper under vacuum. The filtrate obtained was the EM and this was stored at 4°C in a refrigerator.

### Immunocytochemical Staining for the Observation of D38 Infection

Immunocytochemical staining procedure of the root tissue was performed as originally described by Störkuhl et al. (1994). Briefly, fresh *S. miltiorrhiza* hairy root tissues were fixed in 4% paraformaldehyde for more than 24 h. The hairy root tissues were subsequently repaired in the fume hood with a scalpel and then the tissues and the corresponding label were put in the dehydration box. Next, the dehydration box was put into the basket and dehydrated with gradient alcohol in turn. After that, waxed tissue was placed in the embedding machine and subjected to minor modification. Finally, waxed tissue was sliced in paraffin section of 4 μm. The slice was then put into dimethylbenzene xylene I for 15 min, dimethylbenzene xylene II for 15 min, ethanol I for 5 min, anhydrous ethanol II for 5 min, 75% alcohol for 5 min, 85% alcohol for 5 min, and distilled water in turn. And then, the slice was put into the repair box which was filled with EDTA antigen repair buffer (pH 8.0) (Servicebio, Wuhan, China). The antigen repair was performed in the microwave with the medium boiling for 5 min. After natural cooling, the slice was placed into the PBS (pH 7.4) and washed on the decoloring table for three times, 5 min each time. After spin-dry, drew a circle on the slice with Pap Pen to prevent the antibody from running away. Dropping 3% BSA (Solarbio) in the circle, the tissue was covered and closed for 30 min at room temperature. After added with primary antibody [1:100 concanavalin A (GenBank ID, 72333; Sigma) labeled with FITC fluorescence], the slice was placed flat on the wet box with some water and incubated for 24 h at 4°C. Then the slice was put into PBS (pH 7.4) for decoloration (washing three times, 5 min each time). After spin-dry, the slice was added with DAPI (Servicebio, Wuhan, China) and incubated for 10 min at room temperature in dark. After washed for three times and sealed with Antifade Mounting Medium (Servicebio, Wuhan, China), the slice was inverted in the fluorescence microscope to collect images (Nasr et al., 2006).

### Manufacture of D38 Fertilizer

There are 20 g glucose, 3 g KH_2_PO_4_, and 1.5 g MgSO_4_⋅7 H_2_O dissolved in every 1 L deionized water, which was then blended with 250 g wheat bran and 250 g cottonseed shell. After sterilization, this matrix was used as medium for endophyte D38 culture in dark environment. After cultured for 14 days, the number of D38 spores was determined as 1∼2 × 10^9^/g by hemacytometry method, and the matrix was then used as D38 fertilizer. Subsequently, 20 g D38 fertilizers were applied into each *S. miltiorrhiza* aseptic seedling cultivated in sterile soil, whereas the control was added by 20 g blank wheat bran-cottonseed medium in July, 2016. And then the seedlings were cultured under 16h light and 8h dark regime every day. The cultured potted soil matrix is made of pearl cotton, humus and vermiculite with the ratio of 1:3: l. Each treatment has five repeats and each group was sampled every 2 months from September, 2016 to March, 2017 for the measurement of morphological indexes, biomass and bioactive substances content.

### HPLC Analysis

*Salvia miltiorrhiza* hairy roots were desiccated at 55°C in the oven and then grinded into powder. After screened by a 40 mesh, hairy root powder was weighed 0.2 g accurately and put into 10 mL centrifuge tube. Accurately draw 4 mL methanol using 5 mL pipette and add it into the centrifugal tube. After ultrasonically extracted for 60 min, the sample solution was filtered with 0.45 μm syringe filter and subsequently used for HPLC analysis. The analysis was performed on an Agilent-1100 system using a ZOBAX-EXTEND-C18 chromatographic column (250 mm × 4.6 mm, 5 μm) at 30°C with a H_2_O (+0.1% HCOOH) (A)/acetonitrile (B) gradient (0–15 min: 80% B; 15–16 min: 80% B-62% B; 16–25 min: 62% B) as previously described (Ming et al., 2013). Rosmarinic acid, salvianolic acid B, dihydrotanshinone I, tanshinnone I, cryptotanshinone, and tanshinone IIA in the methanol extract of hairy roots were identified by comparison with the available standards. The reference standards were acquired from Chengdu Mansite Pharmacetical Co. Ltd. [Chengdu (Sichuan Province), PR China].

### RNA Isolation and Real-Time Quantitative PCR Analysis

Hairy roots were divided randomly into control group and D38 groups, which were cultured for 18 days and nutrient solution was changed every 6 days. Hairy roots of each group were sampled at 0, 6, 12, and 18 days and preserved in -80°C refrigerator. The total RNA of samples were extracted with mirVana^TM^ miRNA isolation Kit (TaKaRa). After detecting the good integrity of total RNA and obtaining cDNA by reversed transcription, the reaction was started on fluorescence quantitative PCR instrument (LightCycler II 480). The primers used in the real-time PCR reaction is listed in **Table 1** for detecting the gene expressions of *smHMGR* (3-hydroxy-3-methylglutaryl-CoA reductase), *smDXR* (1-deoxyd-xylulose 5-phosphate reductoisomerase), *smGGPPS* (geranylgeranyl diphosphate synthase), *smCPS* (copalyl diphosphate synthase), and *smKSL* (kaurene synthase-like), respectively (Ma et al., 2012). The 18S gene was used as a reference gene to normalize cDNA. The process of RT-PCR reaction is set as follows: initial degeneration at 95°C for 15 min, subsequently 40 cycles of 95°C degeneration for 10 s, and finally 60°C annealing extending for 30 s. The specificity of product was detected by using the melting curve, in which temperature was elevated slowly from 60°C to 97°C, and five fluorescent images were gathered every °C. The relative gene expression was quantified using the comparative CT method.

**Table 1:** Primers used in real time-PCR.

Gene name	Primer
HMGRKF(5′-3′)	AGGCTTTGCAGCGGATAA
HMGRKR(5′-3′)	GAATCTGCACGTATCCCAC
DXRKF (5′-3′)	CCATGACCGGAGTTCTTAG
DXRKR (5′-3′)	GGATGATCTCCTCCAACG
GGPPSKF(5′-3′)	CGAGAAGCTCAACGAGGA
GGPPSKR(5′-3′)	GTTCTGCCTATGTGCAATGTA
CPSKF (5′-3′)	TGCGAAGAGATTCGCCTAC
CPSKR (5′-3′)	CTTGATCTCATCAGGCAAGT
KSLKF (5′-3′)	CATGTCGAACAAGGACGTA
KSLKR (5′-3′)	AATCATCCAAGGTTAGTGCC
18SF (5′-3′)	CCAGGTCCAGACATAGTAAG
18SR (5′-3′)	GTACAAAGGGCAGGGACGTA


### Data Analysis

The data of HPLC analysis and semi-quantitative real-time PCR of hairy root cultures were analyzed by one-way analysis of variance (ANOVA) through SPASS software, with both control and different treatments in triplicate. The results are presented as mean ± SD (standard deviation) and the error bars represent the standard deviation of biological triplicates in the figures. Otherwise, the term significant has been used to denote the differences for which *p* is <0.05 and the statistical significance of differences in gene transcripts was analyzed by one-sample *t*-test.

## Results

### Isolation and Identification of Endophytic Fungus D38

**FIGURE 1:**
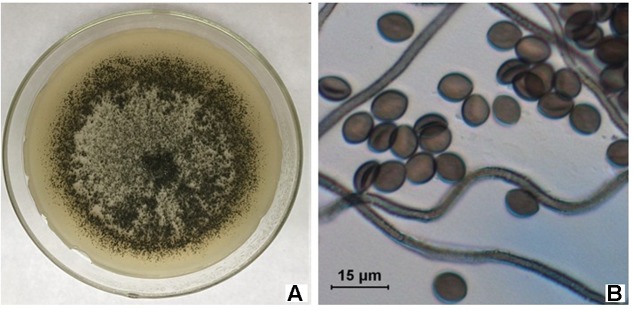
Morphological characteristics and microstructure of D38. **(A)** D38 Colony in PDA; **(B)** Micrograph of D38.

**FIGURE 2:**
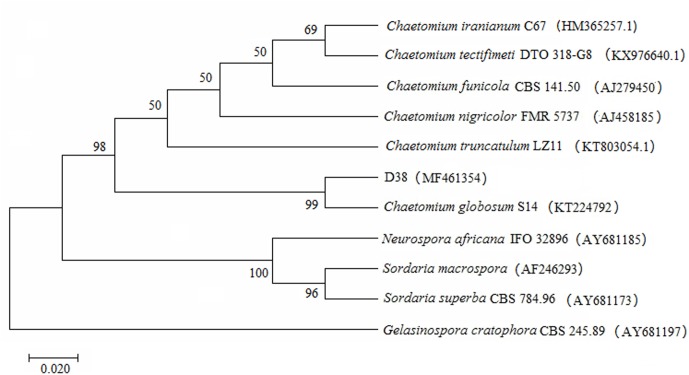
Phylogenetic tree characterization of endophytic fungus D38. The tree presented is a neighbor-joining tree based on ITS gene sequence alignment, which showed the relationship between D38 and other fungi. The ITS gene sequence of D38 shared high homology with *Chaetomium globosum*. The tree was constructed with MEGA7 and 1000 bootstrap replicates based on Kimura 2-parameter model. And *Gelasinospora cratophora* (AY681197) is used as outgrout.

A total of 123 isolates of endophytes were isolated from the roots, stems and leave of *S. miltiorrhiza*. D38 was purified from the roots and its appearance on solid culture is characterized by villous hyphae with yellowish color on the back and numerous dark-brown hairy perithecia (**Figure 1A**). The microstructural features reveal that D38 is long chain without branches and the brown ascospores are lemon-shaped, smooth-surfaced with apical papillae (**Figure 1B**). Subsequently, another isolate D68 from stems displayed the same characteristics as D38. So we combined this two isolates as D38. In this study, the internal transcribed spacer region (ITS) of D38 was selected and sequenced with nuclear ribosomal DNA as a template. Results of the BLAST searches for D38 strain showed that the ITS rDNA sequences of this isolate shared high homology with *Chaetomium globosum* (**Figure 2**). According to the molecular data, the strain D38 was identified to be *Chaetomium globosum*.

### Effects of D38 on the Contents of Tanshinones in *S. miltiorrhiza* Hairy Roots

**FIGURE 3:**
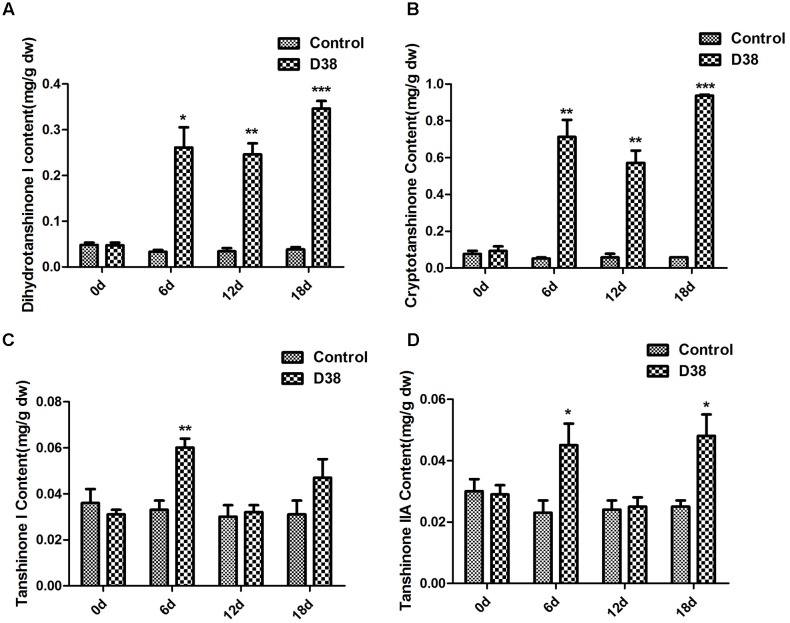
The effect of D38 hyphae on the contents of tanshinones of *S. miltiorrhiza* hairy roots. **(A)** Dihydrotanshinone I content; **(B)** Cryptotanshinone content; **(C)** Tanshinone I content; **(D)** Tanshinone IIA content. Values are presented as means ± SD, *n* = 3. ^∗^*p* < 0.05; ^∗∗^*p* < 0.01; ^∗∗∗^*p* < 0.001 versus the control culture.

During our preliminary study, endophyte D38 can be long-term co-cultured with *S. miltiorrhiza* hairy roots. Further we investigated the effects of the live fungus D38 on tanshinones biosynthesis in *S. miltiorrhiza* hairy roots. As shown in **Figure 1**, administration of D38 significantly enhanced the contents of dihydrotanshinone I (DT-I) and cryptotanshinone (CT) on day 6, 12, and 18, compared with that of the control group. The contents of dihydrotanshinone I and cryptotanshinone were increased by 8 fold and 14.9-fold, respectively, on day 18 (**Figures 3A,B**). However, no such notable activity were observed for D38 on the contents of tanshinone I (T-I) and tanshinone IIA (T-IIA). D38 only increased the content of tanshinone I on day 6 (1.8 fold vs. control) (**Figure 3C**) and elevated the content of tanshinone II A on day 6 (1.7 fold vs. control) and 18 (1.9 fold vs. control) (**Figure 3D**).

### Immunocytochemical Staining of D38 in *S. miltiorrhiza* Hairy Roots

**FIGURE 4:**
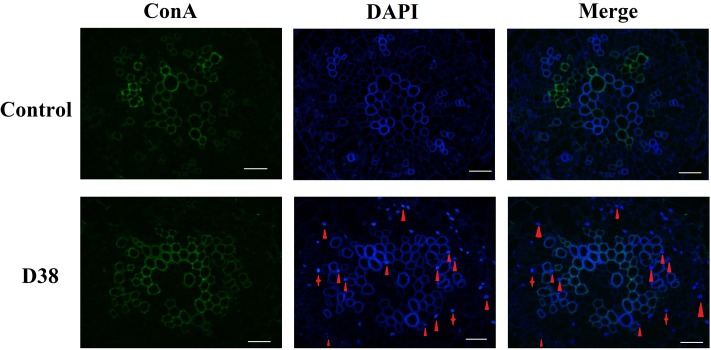
The transection of endophytic fungus D38 localization in *S. miltiorrhiza* hairy roots after immunofluorescence staining. Green: ConA-FITC; Blue: DAPI. Red triangle indicated endophytic fungus D38 located in root intercellular space, while red shuriken indicated endophytic fungus D38 located in root cell (magnification 400×).

After 18 days co-cultivation of *S. miltiorrhiza* hairy root with endophytic fungi D38, the immunofluorescence staining experiment was performed to display the infection locations of D38. As shown in **Figure 4**, endophytic fungi D38 mainly located at the intercellular space or cell junction (red triangle) of *S. miltiorrhiza* hairy root tissue. And minority of D38 hyphal existed in the tissue cells (red shuriken). Thus we suggested that the mycelium of endophytic fungi D38 non-invasively and stably interacted with the host mainly in the intercellular space and formed a non-aggressive symbiosis pattern with the host.

### Effects of Extract of D38 Mycelium (EM) on the Accumulation of Tanshinones in *S. miltiorrhiza* Hairy Roots

**FIGURE 5:**
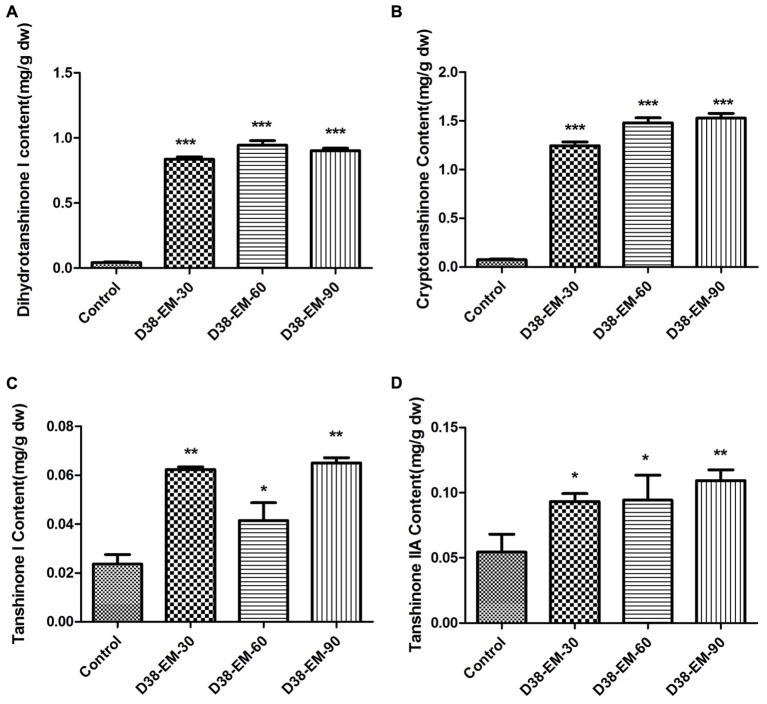
Effects of the extract from D38 mycelium (EM) on the contents of tanshinones in *S. miltiorrhiza* hairy roots. **(A)** Dihydrotanshinone I content; **(B)** Cryptotanshinone content; **(C)** Tanshinone I content; **(D)** Tanshinone IIA content. Values are presented as means ± SD, *n* = 3. ^∗^*p* < 0.05; ^∗∗^*p* < 0.01; ^∗∗∗^*p* < 0.001 versus the control culture.

After 18 days of treatment by different concentration of D38 EM, *S. miltiorrhiza* hairy roots were extracted ultrasonically with methanol for HPLC analysis. The data revealed that D38 EM significantly increased the contents of tanshinones (**Figure 5**). And the most significant increase was observed for the contents of dihydrotanshinone I and cryptotanshinone. The content of dihydrotanshinone I reached the highest level under the action of D38 EM at the dose of 60 mg/L, which was enhanced by 21-fold compared with the control group, while the content of cryptotanshinone reached the highest level under the action of D38 EM at the dose of 90 mg/L, which was increased by 19.8-fold compared with the untreated group. In addition, the contents of tanshinone I and tanshinone IIA were also increased by administration of D38 EM. The content of tanshinone I reached the highest level at 2.2 fold of the control, while the content of tanshinone IIA reached the peak at 2.0 fold of the control, under the action of D38 EM at the dose of 90 mg/L.

### Transcriptional Response of Tanshinone Biosynthetic Pathway to the Induction of D38 EM in *S. miltiorrhiza* Hairy Roots

After RNA extraction and cDNA preparation through the reverse transcription reaction, the transcription levels of five key enzyme genes in the tanshinone biosynthesis pathway were determined by real-time quantitative PCR.

**FIGURE 6:**
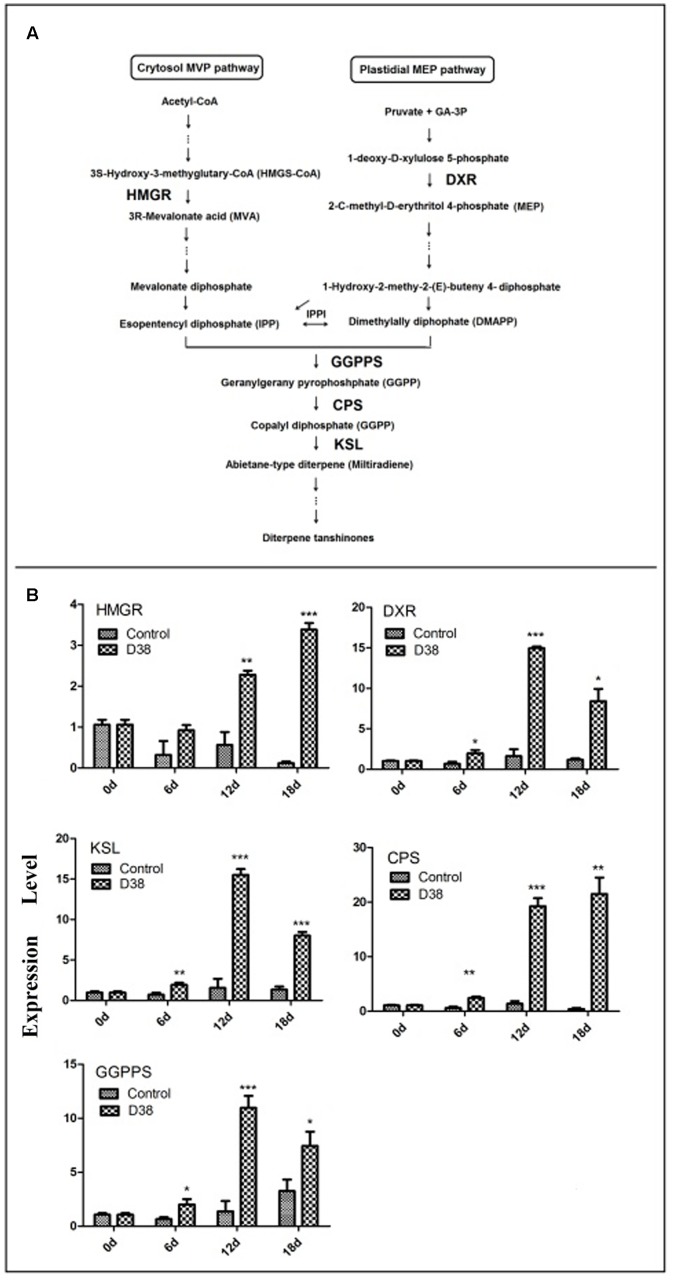
Proposed pathways of tanshinone biosynthesis in *S. miltiorrhiza* **(A)** and effects of D38 EM (100 mg⋅l^-1^) on the expression of genes in tanshinone biosynthetic pathway in *S. miltiorrhiza* hairy roots **(B)**. HMGR, hydroxymethylglutaryl-CoA reductase; DXR, 1-deoxy-d-xylulose 5-phosphate reductoisomerase; GGPPS, geranylgeranyl diphosphate synthase; CPS, copalyl diphosphate synthase; KSL, kaurene synthase-like. Values are presented as means ± SD, *n* = 3. ^∗^*p* < 0.05; ^∗∗^*p* < 0.01; ^∗∗∗^*p* < 0.001 versus the control culture.

As shown in **Figure 6**, D38 EM activated tanshinones biosynthesis and the expression levels of five key enzyme genes were significantly increased. The expression level of 3-hydroxy-3-methylglutaryl-CoA reductase (HMGR), involved in the mevalonate (MVA) pathway, was increased to the highest level at 36-fold of the control samples on day 18. In the 2-*C*-methyl-D-erythritol-4-phosphate (MEP) pathway, the expression level of 1-deoxyd-xylulose 5-phosphate reductoisomerase (DXR) reached the peak of 9.35 fold compared with the control group on day 12. In the downstream pathway, the expression levels of geranylgeranyl diphosphate synthase (GGPPS), copalyl diphosphate synthase (CPS) and kaurene synthase-like (KSL) were all significantly enhanced, especially on day 12 and 18. The expressions of GGPPS and KSL were increased to the highest level at 8 and 10-fold of the control samples respectively on day 12, while the expression of CPS reached the highest expression level at 55.1 fold of the control on day 18.

### Effects of D38 Fertilizer on Plant Growth and Secondary Metabolism in *S. miltiorrhiza*

**FIGURE 7:**
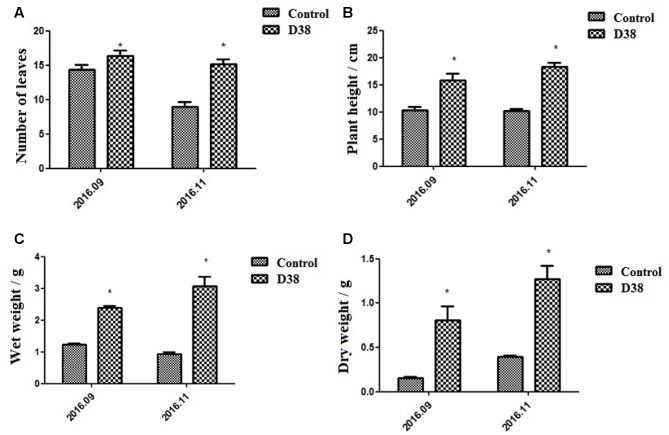
Morphological indexes under the treatment of 20 g D38 fertilizer compared with untreated group from September 2016 to December 2016. **(A)** Number of leaves; **(B)** Plant height; **(C)** Wet weight; **(D)** Dry weight.

**FIGURE 8:**
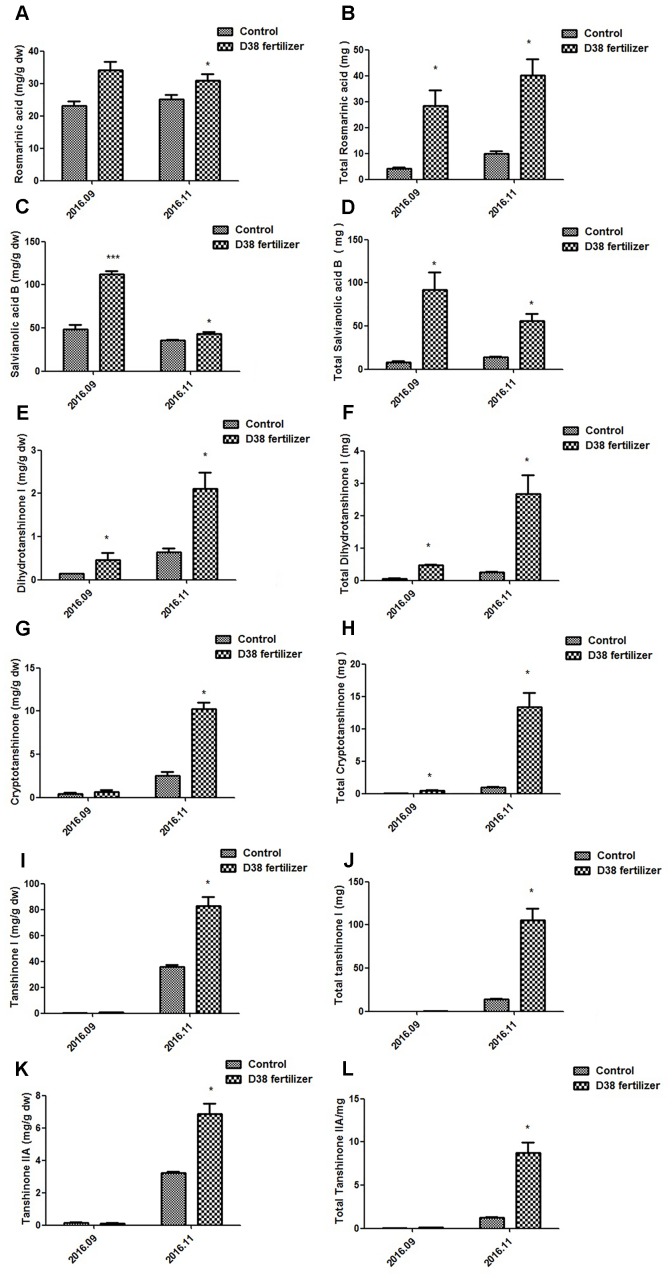
Contents of bioactive substances in *S. miltiorrhiza* root under the treatment of 20 g D38 fertilizer compared with untreated group from September 2016 to December 2016. **(A)** Rosmarinic content per unit of root mass; **(B)** Total rosmarinic content; **(C)** Salvianolic acid B content per unit of root mass; **(D)** Total salvianolic acid B content; **(E)** Dihydrotanshinone I content per unit of root mass; **(F)** Total dihydrotanshinone I content; **(G)** Cryptotanshinone content per unit of root mass; **(H)** Total cryptotanshinone content; **(I)** Tanshinone I content per unit of root mass; **(J)** Total tanshinone I content; **(K)** Tanshinone IIA content per unit of root mass; **(L)** Total tanshinone IIA content.

*Salvia miltiorrhiza* seedlings were co-cultured with 20 g D38 fertilizer for half a year from July 2016 to December 2016. The morphology indexes of *S. miltiorrhiza* were recorded every 2 months from Aug 2016, including the number of leaves, plant height, wet weight and dry weight. At the same time, *S. miltiorrhiza* roots were harvested and then ultrasonically extracted with methanol for HPLC analysis. The data revealed that D38 fertilizer could greatly promote the plant growth of *S. miltoirrhiza* (**Supplementary Figure S1**) and enhance the contents of salvianolic acids and tanshinones (**Figures 7**, **8**).

The plant height, root wet weight and dry weight of D38-treated samples increased stably and were superior to those control samples during the experiment period. Due to some fallen leaves, the number of leaves of both groups was slightly decreased in November 2016 compared with that in September 2016. Those plants of D38 group were much taller than those of the untreated group. Furthermore, the wet weight and dry weight of D38-treated plant roots was 1.95 and 5.20 fold of the control samples, respectively, in November 2016. Our results indicated that D38 was a beneficial endophyte, and can greatly enhance the biomass and facilitate the growth of *S. miltoirrhiza* plants.

D38 fertilizer also stimulated the secondary metabolism in *S. miltoirrhiza*, which significantly enhanced the phenolic acids and tanshinones content per unit of root mass in November, 2016 and the total content of these bioactive substances increased stably during the half year. The rosmarinic acid and salvianolic acid B content per unit of D38 fertilizer treatment was 24% and 21% higher than the control in Nov. 2016, respectively. And the contents of dihydrotanshinone I, cryptotanshinone, tanshinone I and tanshinone IIA per unit were dramatically enhanced by 2.29, 3.07, 1.30, and 1.14 fold in November 2016, respectively. Our results indicated that D38 could be developed into new biotic fertilizer for the promotion of plant growth and secondary metabolite accumulation.

## Discussion

Plants are associated with various kinds of environmental microbes, including pathogens, rhizosphere bacteria, mycorrhizal fungi, and endophytes (Crawford et al., 2010). In natural ecosystems, though most plants are infested by a group of fungi that will not cause obvious disease symptoms, these fungi may trigger a complex network of reactions leading to the biosynthesis and accumulation of secondary metabolites (Zhao et al., 2010). For example, an endophytic fungus, *Aspergillus niger*, isolated from the bark of *Taxus chinensis* tree, could elicit a more than twofold increase in the taxol yield and about a six-fold increase in total secretion in a *T. chinensis* cell suspension culture (Wang et al., 2001). Although fungal endophytes could act as a zoetic elicitor to multiply and persistently stimulate the plant tissue (Saikkonen et al., 2004; Rusch, 2016), there are few reported endophytes from the host plant can be co-cultured with their host plant tissue for a long time. Our study is therefore the first report on the effects of a live endophytic fungus D38 and its elicitor on the tanshinones production in *S. miltiorrhiza* hairy root.

Many factors may affect the results of those plant-microbe interactions, including biotic and abiotic environmental factors, the genotype of the host and the interacting microorganism (Brader et al., 2017). Endophytes may experience a long-term evolution and interactive communication to form a symbiotic relationship with their plants (Wawra et al., 2016). It was reported that endophytes of successful infection may overcome the barrier of root periderm and enter the above-ground part of plant via central cylinder (Sesma and Osbourn, 2004; Sukno et al., 2008). D38 was also separated from the stems of *S. miltiorrhiza*, which indicated it may transfer into the above-ground of plant also by central cylinder and form bitrophic interactions with host not only in roots but also in stems. With respect to the process of endophytes infection, their hyphae may invade into epidemic cell and subsequently develop and form intercellular hyphae, after which the intact host plasma membrane may be beset with intracellular hyphae just for the sake of longer interaction in cortical cells (Kei et al., 2016). Our results showed that the mycelium of endophytic fungi D38 may develop a noninvasive pattern by mainly colonizing and residing in the intercellular gap of xylem parenchyma cells of *S. miltiorrhiza* hairy root, verifying a stable and beneficial relationship between the endophyte and host.

In our study, the accumulation of tanshinones in *S. miltiorrhiza* hairy roots was enhanced by both endophytic *Chaetomium globosum* D38 and its EM, which indicated that D38 could significantly stimulate the secondary metabolism in *S. miltiorrhiza* (**Figures 3**, **5**). As far as we know, the effects of endophytic fungi and its elicitors on the secondary metabolism of their host plants, especially medicinal plants, were rarely reported. Fungal endophyte infection was showed to change the metabolic profiles of *Lolium perenne* by regulating carbon/nitrogen exchange (Rasmussen et al., 2008). Also our group has previously reported an elicitor from endophyte *Trichoderma atroviride* D16, which could significantly promote the biosynthesis of tanshinone constituents (Ming et al., 2013). However, the hairy roots of *S. miltiorrhiza* can not be long-term co-cultured with D16. So our present work is the first report on the effects of a live endophytic fungus D38 on the tanshinones production in *S. miltiorrhiza* hairy roots.

A previous report indicated that tanshinones exhibited stronger antimicrobial activity than phenolic acids, among which DT-I and CT exhibited much better activities (Zhao et al., 2011). This may explain that why D38 and its EM could induce higher levels of DT-I and CT biosynthesis than those of other compounds (**Figure 5**). The phenomenon could be interpreted that *S. miltiorrhiza* hairy roots may produce defensive response to the invasion of *C. globosum* D38 mycelium or elicitors through secreting more DT-I and CT to protect itself.

Tanshinones were one kind of bioactive abietanoid diterpenes in *S. miltiorrhiza* roots, owning versatile therapeutic effects. Nevertheless, these components account for a low proportion in *S. miltiorrhiza* and are regulated by a series of rate-limiting enzymes (HMGR, DXR, GGPPS, CPS, and KSL) (**Figure 6**). Isopentenyl diphosphate (IPP) and dimethylallyl diphosphate (DMAPP) are general precursors of terpenoids in plants and synthesized via mevalonate (MVA) and 2-*C*-methyl-D-erythritol phosphate (MEP) pathway. MVA pathway proceeds in the cytoplasm, while MEP pathway carries out in the plastids. HMGR is one of most significant enzymes in the MVA pathway, while DXR is very important in the MEP pathway. GGPPS is the junction enzyme between MVA and MEP pathway. As to the downstream of tanshinone biosynthesis, CPS and KSL are two key points as we know. By comparing the effects of *C. globosum* with EM, we found that EM exhibited better activity in promoting tanshinones biosynthesis in *S. miltiorrhiza* hairy root cultures. So we further investigated the effects of EM on the transcriptional levels of above-mentioned five selected key genes by real-time quantitative PCR. As shown in **Figure 6**, from day 6 to day 12, the transcriptional levels of the five tested genes were all elevated gradually and significantly compared to those of control, whereas a little decline in the expressions of GGPPS, CPS, and KSL were observed in EM-treated group on day 18. These results showed that EM stimulated key genes expression and induced metabolic flux to tanshinones biosynthesis, which finally led to the accumulation of tanshinones in *S. miltiorrhiza* hairy roots. However, it is still unknown that how EM stimulated these genes through signal transduction, which merits further study regarding the precise site and the mechanism of action.

Additionally, *C. globosum* D38, processed as a biotic fertilizer, was also administrated to *S. miltiorrhiza* seedlings to test whether this live fungus could exert favorable effects on the whole plant so as to lay the foundation for further practical applications. Our results showed that D38 can significantly promote the growth of *S. miltiorrhiza* seedlings and enhance the contents of both phenylpropionic acids and tanshinones in *S. miltiorrhiza* root, which may lead to a practical breakthrough in *S. miltiorrhiza* cultivation. As we all know, infection by the fungal endophyte may affect the accumulation of nutrition in the host, such as inorganic and organic N (Bethlenfalvay et al., 1982; Lyons et al., 1990). So we suggested that, in soil environment, D38 could help the host make better use of nutrition and enhance the primary metabolism fluxes, which finally led to the fully stimulation of plant growth and secondary metabolism.

## Conclusion

We obtained a beneficial endophyte, *Chaetomium globosum* D38, from the roots of *S. miltiorrhiza*. D38 and its EM could elicit defense responses in *S. miltiorrhiza* hairy roots so as to increase the production of antimicrobial tanshinones of the host by enhancing the expression of related key genes involved in tanshinones biosynthesis. EM exhibited better activity than live fungus D38 and thus can be used as a potent elicitor and convenient strategy for the extensive production of tanshinones in *S. miltiorrhiza* hairy root culture system. Though EM showed stronger effect than the live endophytic fungus on tanshinones synthesis in hairy root cultures, we found that live D38 can be long-term co-cultured with *S. miltiorrhiza* hairy root and therefore suggested that the treatment of live *C. globosum* D38 may be more convenient and feasible in plant cultivation even with more comprehensive and durable effects. All these were proven by our subsequent results that D38 fertilizer can significantly promote the growth of *S. miltiorrhiza* seedlings and enhance the contents of both phenylpropionic acids and tanshinones in *S. miltiorrhiza* roots, which indicated that *Chaetomium globosum* D38 was a highly beneficial endophytic fungus for the growth and metabolism in *S. miltiorrhiza* cultivation.

## Author Contributions

CZ and LQ conceived and designed the experiments. XZ, DL, and XL performed the experiments. XZ and XL analyzed the data. XZ and DL wrote the manuscript. TH, ZK, and JJ provided technical assistance to XZ and XL. MJ and KR revised the language of the article.

## Conflict of Interest Statement

The authors declare that the research was conducted in the absence of any commercial or financial relationships that could be construed as a potential conflict of interest.

## Supplementary Material

**FIGURE S1** | Figures of *S. miltiorrhiza* seedlings under the treatment of 20 g D38 fertilizer compared with untreated group from September 2016 to December 2016. **(A)** The *S. miltiorrhiza* seedlings of untreated group in September 2016; **(B)** The *S. miltiorrhiza* seedlings treated by 20 g D38 fertilizer in September 2016; **(C)** The *S. miltiorrhiza* seedlings of untreated group in December 2016; **(D)** The *S. miltiorrhiza* seedlings treated by 20 g D38 fertilizer in December 2016.
