# Supplementary figures and images for: Endophyte Chaetomium globosum D38 Promotes Bioactive Constituents Accumulation and Root Production in Salvia miltiorrhiza
Source: Front Microbiol. 2018 Jan 22;8:2694. doi: 10.3389/fmicb.2017.02694 (PMC5786870; doi:10.3389/fmicb.2017.02694)

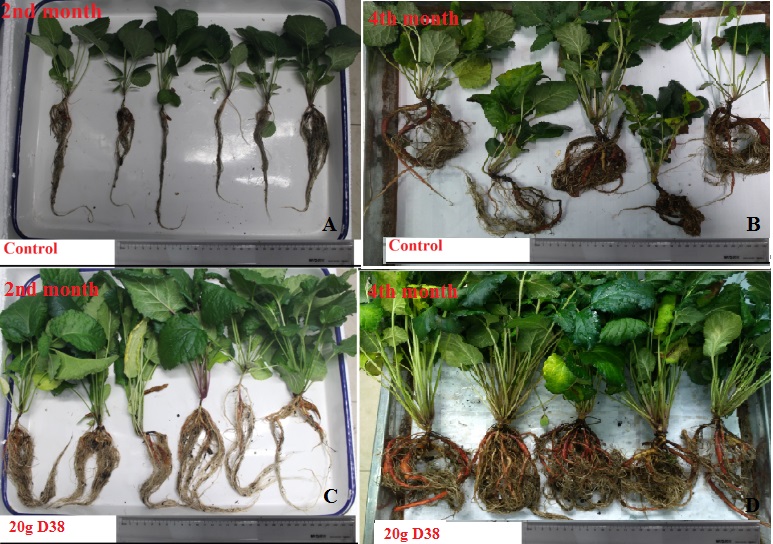

Supplement: Supplementary file 1 [file Image_1.JPEG]
